# Fish Cholesterol 25-Hydroxylase Inhibits Virus Replication via Regulating Interferon Immune Response or Affecting Virus Entry

**DOI:** 10.3389/fimmu.2019.00322

**Published:** 2019-03-06

**Authors:** Ya Zhang, Liqun Wang, Xiaohong Huang, Shaowen Wang, Youhua Huang, Qiwei Qin

**Affiliations:** ^1^College of Marine Sciences, South China Agricultural University, Guangzhou, China; ^2^Key Laboratory of Tropical Marine Bio-resources and Ecology, South China Sea Institute of Oceanology, Chinese Academy of Sciences, Guangzhou, China; ^3^University of Chinese Academy of Sciences, Beijing, China; ^4^Laboratory for Marine Biology and Biotechnology, Qingdao National Laboratory for Marine Science and Technology, Qingdao, China

**Keywords:** cholesterol 25-hydroxylase, grouper, Singapore grouper iridovirus, red-spotted grouper nervous necrosis virus, viral replication, interferon-stimulated gene, viral entry

## Abstract

Cholesterol 25-hydroxylase (CH25H) is an interferon (IFN)-induced gene that catalyzes the oxidation of cholesterol to 25-hydroxycholesterol (25HC), which exerts broad-spectrum antiviral function. To investigate the roles of fish CH25H in Singapore grouper iridovirus (SGIV) and red-spotted grouper nervous necrosis virus (RGNNV) infection, we cloned and characterized a CH25H homolog from orange-spotted grouper (*Epinephelus coioides*) (EcCH25H). EcCH25H encoded a 271-amino-acid polypeptide, with 86 and 59% homology with yellow croaker (*Larimichthys crocea*) and humans, respectively. EcCH25H contained a conserved fatty acid (FA) hydroxylase domain and an ERG3 domain. EcCH25H expression was induced by RGNNV or SGIV infection, lipopolysaccharide (LPS) or poly (I:C) treatment *in vitro*. Subcellular localization showed that EcCH25H and mutant EcCH25H-M were distributed in the cytoplasm and partly colocalized with the endoplasmic reticulum. SGIV and RGNNV replication was decreased by EcCH25H overexpression, which was reflected in the reduced severity of the cytopathic effect and a decrease in viral gene transcription, but replication of both viruses was increased by knockdown of EcCH25H. Besides, the antiviral activity was dependent on its enzymatic activity. Treatment with 25HC significantly inhibited replication of SGIV and RGNNV. EcCH25H overexpression positively regulated the IFN-related molecules and proinflammatory cytokines, and increased both IFN and ISRE promoter activities. Moreover, 25HC treatment significantly suppressed SGIV and RGNNV entry into host cells. The similar inhibitory effect on SGIV entry was observed in EcCH25H overexpression cells. Taken together, our findings demonstrated that EcCH25H inhibited SGIV and RGNNV infection by regulating IFN signaling molecules, and might also influence viral entry via an effect on cholesterol.

## Introduction

The innate immune response is the first line of defense against invading pathogens, by which numerous pattern recognition receptors (PRRs) recognize pathogens, triggering the promotion of the interferon (IFN)-mediated innate immune response and leading to transcription of numerous IFN-stimulated genes (ISGs) (1–3). It has been reported that ISGs exert antiviral effects by inhibiting specific stages in the life cycle of viruses (4). For example, IFN-induced transmembrane proteins (IFITMs) block viral entry (5, 6), and protein kinase R (PKR) inhibits translation of some viral proteins through the suppression of eukaryotic translation initiation factor (eIF)2a elongation factors (7), while viperin and tetherin inhibit virus release from host cells (8, 9). Cholesterol 25-hydroxylase (CH25H), an ISG (10, 11), is an endoplasmic reticulum (ER)-associated membrane protein that catalyzes the conversion of cholesterol to 25-hydroxycholesterol (25HC) to reduce cholesterol accumulation (12, 13). 25HC, a natural oxysterol, plays a role in regulating cholesterol homeostasis and sterol biosynthesis through the regulation of nuclear receptors and sterol response element–binding proteins (SREBPs; in particular SREBP2) (12, 14, 15). Recent reports have revealed that 25HC inhibits the replication of various enveloped viruses, including hepatitis C virus (HCV) (16, 17), porcine reproductive and respiratory syndrome virus (PRRSV) (18), murine cytomegalovirus (MCMV) (19), West Nile virus (20), pseudorabies virus (PRV) (21), herpes simplex virus 1 (22), as well as one non-enveloped virus (poliovirus) (23), but it is inactive against another non-enveloped virus (adenovirus) (19). Besides, increased evidence demonstrates that 25HC blocks the entry of some viruses, including vesicular stomatitis virus (VSV) (19), human immunodeficiency virus (HIV) (19) and PRRSV (24), by modifying membrane fusion between viruses and cells.

Groupers, *Epinephelus* spp., are important commercial fish species in China and Southeast Asian countries. In recent years, outbreaks of infections caused by Singapore grouper iridovirus (SGIV) and red spotted grouper nervous necrosis virus (RGNNV) have caused heavy economic losses in the grouper industry (25–27). SGIV, an enveloped double-stranded large DNA virus, was first isolated from diseased grouper (*Epinephelus tauvina*) and characterized as a novel member of the genus *Ranavirus*, family *Iridoviridae* (26, 28). The SGIV genome consists of 140,131 bp, encoding 162 open reading frames (ORFs) (29). RGNNV is a non-enveloped icosahedral RNA virus, belonging to the genus *Betanodavirus*, family *Nodavirdae*, which has been frequently isolated from grouper and European sea bass (*Dicenthrarchus labrax*). The RGNNV genome is composed of two single-stranded positive-sense RNAs (30); RNA1 (3.1 kb) encodes the RNA-dependent RNA-polymerase (RdRp), while RNA2 (1.4 kb) encodes the capsid protein (CP). In addition, RNA3, a subgenomic transcript of RNA1, contains one ORF that encodes two non-structural proteins (31).

In order to clarify the mode of action of grouper viruses, a large number of immune genes were cloned and their roles in grouper virus infection were studied. For example, some immune-related genes, such as ISG15 (32), mitochondrial antiviral signaling protein (MAVS) (33), and IFN regulatory factor (IRF) 3 (34) exhibited different antiviral activities against SGIV or RGNNV infection. Notably, the transcriptome analysis of virus-infected grouper spleen showed that several ISGs were regulated in response to SGIV (35), or RGNNV (36), while the roles of most ISGs in fish virus replication remained unclear.

In the present study, a CH25H homolog from orange-spotted grouper (EcCH25H) was cloned and characterized. We investigated the antiviral roles of EcCH25H during the replication of SGIV and RGNNV. In addition, we found that EcCH25H influenced the entry of SGIV and RGNNV. Our data will provide new insights into the function of fish CH25H genes against infection by fish viruses.

## Materials and Methods

### Cells and Viruses

Grouper spleen (GS) cells used in this study were grown in Leibovitz's L15 medium containing 10% fetal bovine serum (FBS; Gibco) at 28°C (37). The virus stocks of SGIV and purified SGIV were propagated in GS cells, while the RGNNV stocks were propagated in grouper brain (GB) cells, and the titers of the viruses were determined in GS cells and GB cells, respectively, that were both grown in Leibovitz's L15 medium containing 10% FBS (32, 38). Virus stocks were maintained at −80°C.

### Reagents

25HC was purchased from Santa Cruz Biotechnology (sc-214091), reconstituted in chloroform to a concentration of 12.4 mM, and stored at −20°C. Poly (I:C) and lipopolysaccharide (LPS) were purchased from Sigma–Aldrich. Poly (I:C) was reconstituted in RNase-free water at a concentration of 5 mg/mL and LPS was reconstituted in phosphate-buffer saline (PBS) at a concentration of 2.5 mg/mL. The lipophilic dye DiO and amine-reactive Cy5 were purchased from Biotium.

### Cloning of EcCH25H and Sequence Analysis

According to the expressed sequence tag (EST) sequences of EcCH25H from the grouper spleen transcriptome (35), the full-length EcCH25H sequence was cloned using the primers listed in the Table 1 by PCR amplification. Sequence analysis of EcCH25H was carried out using the BLAST program (http://www.ncbi.nlm.nih.gov/blast), and the conserved domains were predicted using the SMART program (http://smart.embl-heidelberg.de/). Multiple amino-acid sequence alignments were performed using the ClustalX1.83 software and the data were edited using the GeneDoc program. The phylogenetic tree was carried out using the MEGA 6.0 software.

**Table 1:** Primers used in this study.

**Primer names**	**Sequence (5^**′**^-3^**′**^)**
EcCH25H-ORF-F	ATGAATGTGCTCTCCACAGAG
EcCH25H-ORF-R	CTCTGGTCGTAAATACTGTGCTGAT
EcCH25H-C1-EcorI-F	CCGGAATTCTATGAATGTGCTCTCCACAGAG
EcCH25H-C1-BamHI-R	CGCGGATCCCTCTGGTCGTAAATACTGTGCTGAT
EcCH25H-M-F	CAAGCCCAACAGTCTCTCCACAATGTCAACTACGCCCCCTAC
EcCH25H-M-R	GTGGAGAGACTGTTGGGCTTGGTGGCAGGGAGCTCC
siRNA1	CCAGAGCAGAGAGAGATCCTGCTTT
siRNA2	ACCGCGCTGTTTCACTTACTGAGAA
siRNA3	GACAGCAGCTCACTGGAGCTGTTAT
EcCH25H-RT-F	TCCACAGAGCATGTAGAGGG
EcCH25H-RT-R	GTAGGACGCAGGCAGGTAA
Actin-RT-F	TACGAGCTGCCTGACGGACA
Actin-RT-R	GGCTGTGATCTCCTTCTGCA
RGNNV CP-RT-F	CAACTGACAACGATCACACCTTC
RGNNV CP-RT-R	CAATCGAACACTCCAGCGACA
RGNNV RdRp-RT-F	GTGTCCGGAGAGGTTAAGGATG
RGNNV RdRp-RT-R	CTTGAATTGATCAACGGTGAACA
SGIV MCP-RT-F	GCA CGCTTCTCTCACCTTCA
SGIV MCP-RT-R	AACGGCAACGGGAGCACTA
SGIV VP19-RT-F	TCCAAGGGAGAAACTGTAAG
SGIV VP19-RT-R	GGGGTAAGCGTGAAGAC
EcIRF3-RT-F	GACAACAAGAACGACCCTGCTAA
EcIRF3-RT-R	GGGAGTCCGCTTGAAGATAGACA
EcIRF7-RT-F	CAACACCGGATACAACCAAG
EcIRF7-RT-R	GTTCTCAACTGCTACATAGGG
EcISG15-RT-F	CCTATGACATCAAAGCTGACGAGAC
EcISG15-RT-R	GTGCTGTTGGCAGTGACGTTGTAGT
EcIFP35-RT-F	TTCAGATGAGGAGTTCTCTCTTGTG
EcIFP35-RT-R	TCATATCGGTGCTCGTCTACTTTCA
EcMXI-RT-F	CGAAAGTACCGTGGACGAGAA
EcMXI-RT-R	TGTTTGATCTGCTCCTTGACCAT
EcMXII-RT-F	GCTTCATCAACTACAAGACCTTCGA
EcMXII-RT-R	CGCCTTCCTAACAGTATCTCCTATTT
EcTNFα-RT-F	GTGTCCTGCTGTTTGCTTGGTA
EcTNFα-RT-R	CAGTGTCCGACTTGATTAGTGCTT
EcIL-6-RT-F	GGTTGGTCCAAGGTGTGCTTA
EcIL-6-RT-R	CTGGGATTGTCGAGGTCCTT
EcIL-8-RT-F	GCCGTCAGTGAAGGGAGTCTAG
EcIL-8-RT-R	ATCGCAGTGGGAGTTTGCA

### Expression Patterns of EcCH25H in Innate Immunity

To illustrate the expression changes of EcCH25H in response to fish virus infection, GS cells were infected with SGIV or RGNNV at a multiplicity of infection (MOI) of 2.0, and harvested at 4, 8, 18, 24, 36 h post-infection (h.p.i.) for RNA extraction and quantitative real-time PCR (qPCR) analysis. To examine the expression profiles of EcCH25H in response to pathogen-associated molecular pattern (PAMP) molecules, GS cells were transfected with 200 ng poly (I:C) or treated with 4 μg/mL LPS, and collected at 4, 8, 12, 24, 36 h for further qPCR analysis.

### Plasmid Construction and Cell Transfection

To clarify the molecular function of EcCH25H *in vitro*, the full-length wild-type EcCH25H (aa 1–271) sequence was subcloned into pEGFP-C1 using the primers in Table 1. Histidine codons (H) at positions 239 and 240 of wild-type EcCH25H (16) were converted to glutamine codons (Q) by site-directed mutagenesis to create the mutant form (EcCH25H-M) lacking catalytic activity. The constructed plasmids (pEGFP-EcCH25H and pEGFP-EcCH25H-M) were subsequently verified by DNA sequencing.

Cell transfection was carried out using Lipofectamine 2000 reagent (Invitrogen). GS cells were seeded in 24-well plates at 60–70% confluence for 18–24 h, and cells were transfected with the mixture of 2 μL Lipofectamine 2000 and 800 ng plasmids and incubated for 6 h. After replacing with fresh normal medium, cells were cultured at 28°C for further study.

### Subcellular Localization

To determine the subcellular localization of EcCH25H, pEGFP-C1, pEGFP-EcCH25H, and pEGFP-EcCH25H-M were cotransfected with the pDsRed2-ER plasmid into GS cells as described above. At 48 h post-transfection, cells were fixed with 4% paraformaldehyde, and stained with 4,6-diamidino-2-phenylindole (DAPI). Fluorescent images were observed by confocal laser scanning microscopy (CLSM).

### Virus Infection Assay

To demonstrate the roles of CH25H during virus infection, GS cells were manipulated using gene overexpression, RNA interference, or treatment with the product of CH25H, and then infected with fish viruses. In detail, GS cells that were transfected with pEGFP-C1, pEGFP-EcCH25H, or pEGFP-EcCH25H-M for 24 h were infected with SGIV or RGNNV at MOI 2.0. Cell morphology was observed and photographed using a phase contrast microscope; meanwhile, mock- and virus-infected cells were harvested at 24 h.p.i. for RNA extraction and qPCR analysis.

To knockdown the expression level of EcCH25H in GS cells, three small interfering RNAs (siRNAs) targeting different sequences of EcCH25H mRNA were commercially synthesized by Invitrogen. GS cells were grown in 24-well plates and then transfected with siRNAs (Table 1). The knockdown efficiency and transcription of viral genes were measured by qPCR.

In addition, the antiviral effects of metabolic product of CH25H, GS cells were treated with different concentrations of 25HC (1, 2, 4, or 8 μM) and incubated for 72 h. The cell viability was determined by 3-(4,5-dimethylthiazol-2-yl)-2,5-diphenyltetrazolium bromide (MTT) assay. The whole-cell lysates of treated infected cells were collected at the indicated time points and virus titers were determined. GS cells were seeded in 96-well plates for 18–24 h, and then infected with serial 10-fold dilutions of SGIV samples in eight replicates. After 96–144 h.p.i., the 50% tissue culture infective dose (TCID_50_) assay was determined using the Reed–Muench method.

### Immunofluorescence Assay

GS cells were pretreated with different concentrations of 25HC (1, 2, or 4 μM) for 12 h, and infected with RGNNV or SGIV at MOI 2.0. At 24 h.p.i., cells were fixed with 4% paraformaldehyde at 4°C overnight, and permeabilized with PBS containing 0.2% Triton X-100 for 15 min. After washing three times with PBS, the cells were blocked with 2% bovine serum albumin (BSA) (Sigma-Aldrich) for 30 min, and incubated with primary antibodies (anti-CP serum 1:200) diluted in 0.2% BSA for 2 h at room temperature. The cells were washed three times with PBS, and incubated with the secondary antibody, fluorescence isothiocyanate-conjugated goat anti-rabbit IgG (1:200; Pierce) at room temperature. After 2 h, cells were stained with 1 mg/mL DAPI and observed under an inverted fluorescence microscope (Zeiss).

### Reporter Gene Assay

To illustrate the activated patterns of IFN promotion by EcCH25H, luciferase plasmids including ISRE-Luc, IFN1-Luc and IFN3-Luc were used. GS cells were cotransfected with 150 ng ISRE-Luc, IFN1-Luc, and IFN3-Luc, and 400 or 800 ng of either pEGFP-EcCH25H or pEGFP-C1. A total of 40 ng SV40 was included to normalize luciferase activity. At 48 h post-transfection, cells were harvested to measure the luciferase activities using the Dual-Luciferase^®^ Reporter Assay System (Promega), as described in the manufacturer's instructions.

### Virus Entry Assay

Whether EcCH25H affected SGIV and RGNNV entry was investigated using two strategies, namely observation under CLSM and qPCR. On the one hand, GS cells were seeded in glass-bottom cell culture dishes and transfected with pEGFP-EcCH25H for 24 h, or pretreated with 25HC for 12 h. After the indicated times, the cells were prechilled at 4°C for 5 min and infected with Cy5-labeled SGIV at 4°C for 20 min in serum-free medium at 28°C for 1 h. After being washed three times with medium, cells were fixed with 4% paraformaldehyde overnight and stained with the lipophilic dye DiO for 30 min (38). The cells were observed under CLSM and photographed. In each sample, 30 cells were selected for analysis and the data were represented as mean ± SEM.

Given that highly purified RGNNV particles (about 25 nm in diameter) was difficult to be obtained for confocal microscopy, the effects of the 25HC on RGNNV entry were determined using qPCR. In brief, GS cells were pretreated with 25HC for 12 h and infected with RGNNV or SGIV in serum-free medium. After 1 h, cells were washed three times with cold serum-free medium to remove unbound virus. Medium with 25HC was added and the cells were cultured for another 4 h (SGIV) or 1 h (RGNNV) at 28°C. Cells were washed once with medium and treated with 0.2 mL citric acid buffer (citric acid 40 mM, potassium chloride 10 mM, sodium chloride 135 mM, pH 3.0) for 1 min. Cells were harvested for RNA extraction and qPCR analysis to measure the amount of virus that had entered the cells (39).

### RNA Isolation and qPCR

Total RNA was extracted and reversed using the ReverTra Ace qPCR RT Kit (TOYOBO) as described previously (32). qPCR was performed in a Roche 480 Real Time Detection System (Roche). Each assay was carried out under the following cycling conditions: 95°C for 5 min for activation, followed by 45 cycles at 95°C for 5 s, 60°C for 10 s and 72°C for 15 s. The primers are listed in Table 1. The expression level of target genes normalized to β-actin was calculated with the 2^−ΔΔ*CT*^ method. The data were indicated as mean ± SD and were shown from one representative experiment carried out in triplicate. Statistics were carried out using SPSS version 20 (IBM, Armonk, NY, USA) by one-way ANOVA. Differences were considered statistically significant when *P* was < 0.05.

## Results

### Sequence Characterization of EcCH25H

**Figure 1:**
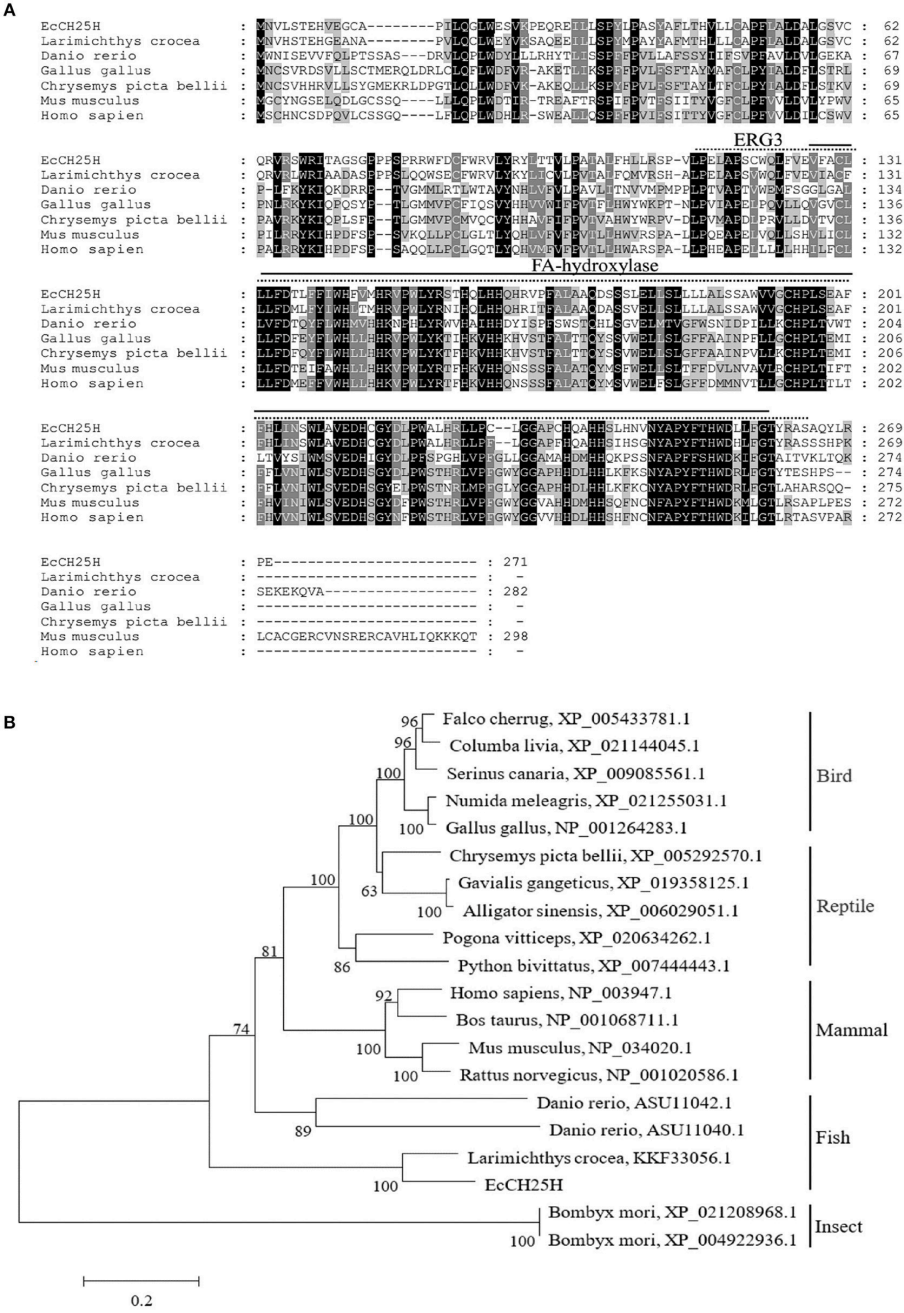
Analysis of CH25H proteins. **(A)** Amino acid alignment of CH25Hs from different species. The ERG3 and FA-hydroxylase domains are represented by straight and dotted lines, respectively. Accession numbers were: *Larimichthys crocea*, KKF33056.1; *Danio rerio*, ASU11040.1; *Gallus gallus*, NP_001264283.1; *Chrysemys picta bellii*, XP_005292570.1; *Mus musculus*, NP_034020.1; *Homo sapiens*, NP_003947.1. **(B)** Phylogenetic analysis of CH25Hs. A neighbor-joining tree was constructed based on the protein sequences of CH25H-like genes from different species using MEGA 6.0 software. Numbers at the nodes denote the bootstrap values of 1,000 replicates. Scale represents the numbers of substitutions per 1,000 bases.

On the basis of the EST sequences from grouper spleen transcriptome, the full-length ORF of EcCH25H was obtained by PCR. EcCH25H encoded a 271-amino-acid polypeptide that shared 86% and 59% identity with the yellow croaker, *Larimichthys crocea* (KKF33056.1) and human, *Homo sapiens* (NP_003947.1), respectively. Amino acid (aa) alignment analysis indicated that EcCH25H contained two conserved domains: ERG3 (aa 113–264) and fatty acid (FA) hydroxylase (aa 127–260) (Figure 1A). Phylogenetic analysis revealed that EcCH25H showed the closest relationship to that of *L. crocea*. All the CH25Hs from different fish species were clustered into one group that was separated from the other groups, including birds, reptiles, mammals, and insects (Figure 1B).

### Expression Profiles of EcCH25H *in vitro*

**Figure 2:**
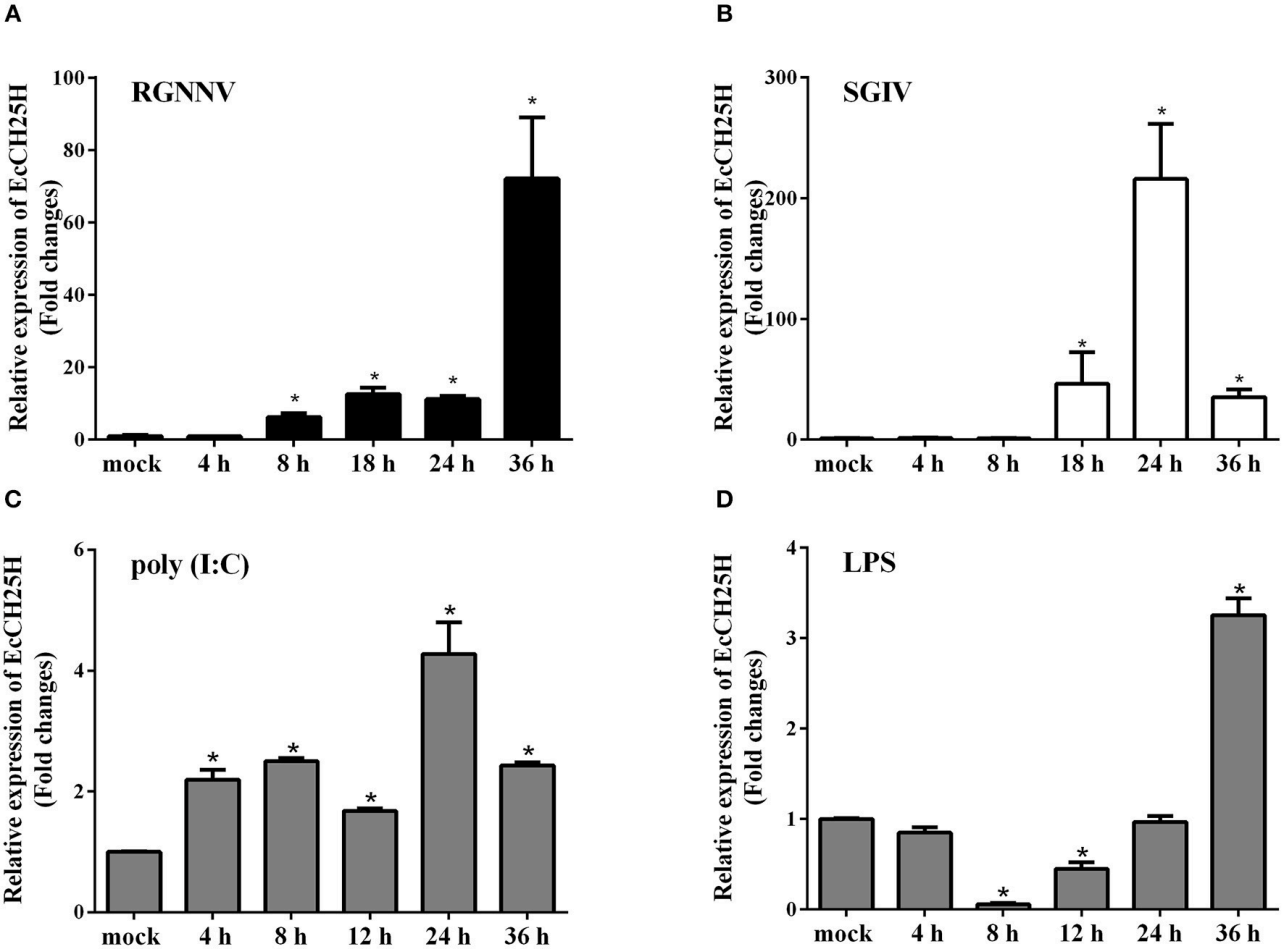
Expression patterns of EcCH25H *in vitro*. GS cells were seeded in 24-well plates overnight, then infected with RGNNV **(A)** or SGIV **(B)** at MOI 2, transfected with 200 ng poly (I:C) **(C)** or pre-treated with 4 μg/mL LPS **(D)** for 36 h. At the indicated time points, GS cells were sampled to detect the expression levels of EcCH25H by qPCR (*n* = 3, means ± SD). ^*^P < 0.05.

To assess the changes in expression of EcCH25H in challenged GS cells, the transcription level of EcCH25H was examined by qPCR after infection with RGNNV or SGIV, or treatment with poly (I:C) or LPS. As demonstrated in Figure 2A, EcCH25H transcription level increased gradually and reached a peak at up to 72-fold higher than of the mock-infected control cells at 36 h during RGNNV infection. During infection with SGIV, EcCH25H transcription level was up-regulated between 18 and 24 h.p.i., reaching 216-fold higher than the level attained in the mock-infected cells, before decreasing at 36 h.p.i. (Figure 2B). After transfection with poly (I:C), EcCH25H expression level was induced gradually and reached a peak of 4.3-fold at 24 h compared to the controls (Figure 2C), while EcCH25H mRNA levels could also be induced by LPS (Figure 2D). Thus, EcCH25H is induced in response to poly (I:C), LPS or fish virus infection.

### Subcellular Localization of EcCH25H

**Figure 3:**
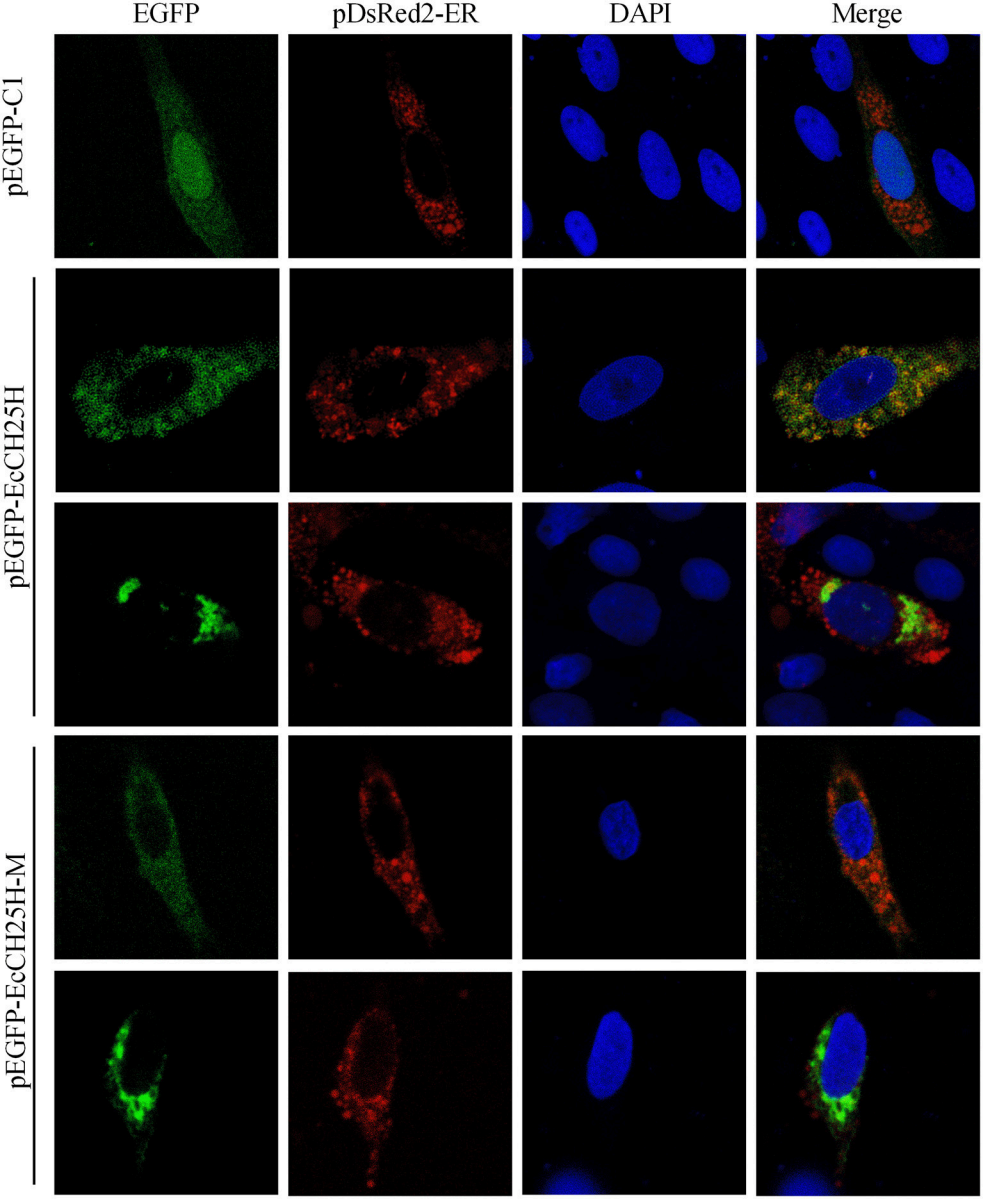
Subcellular localization of EcCH25H in GS cells. PEGFP-C1, pEGFP-EcCH25H, or pEGFP-EcCH25H-M were co-transfected with pDsRed2-ER plasmid into GS cells for 48 h, and stained with DAPI. Samples were observed under CLSM.

CH25H is an ER-associated hydroxylase that catalyzes the conversion of cholesterol to 25HC, and CH25H contains tripartite histidine residues, which are critical for their hydroxylase activities (40). To explore whether EcCH25H was colocalized with ER *in vitro* and whether its localization was dependent on hydroxylase activity, EcCH25H-M was generated by converting histidine codons at positions 239 and 240 to glutamines, using site-directed mutagenesis. For subcellular localization analysis, pEGFP-C1, pEGFP-EcCH25H, or pEGFP-EcCH25H-M, was cotransfected with pDsRed2-ER plasmid into GS cells. The green fluorescence in pEGFP-C1-transfected cells was distributed throughout the cytoplasm and nucleus (Figure 3). However, in pEGFP-EcCH25H- and pEGFP-EcCH25H-M-transfected cells, the green fluorescence was distributed in the cytoplasm in two forms: point-like uniform and dot-like aggregation forms, which were both colocalized partly with the red fluorescence of the ER. Therefore, we suggest that EcCH25H encoded an ER-localized protein, and that its localization was independent of hydroxylase activity.

### Antiviral Effects of EcCH25H on Fish Virus Replication *in vitro*

**Figure 4:**
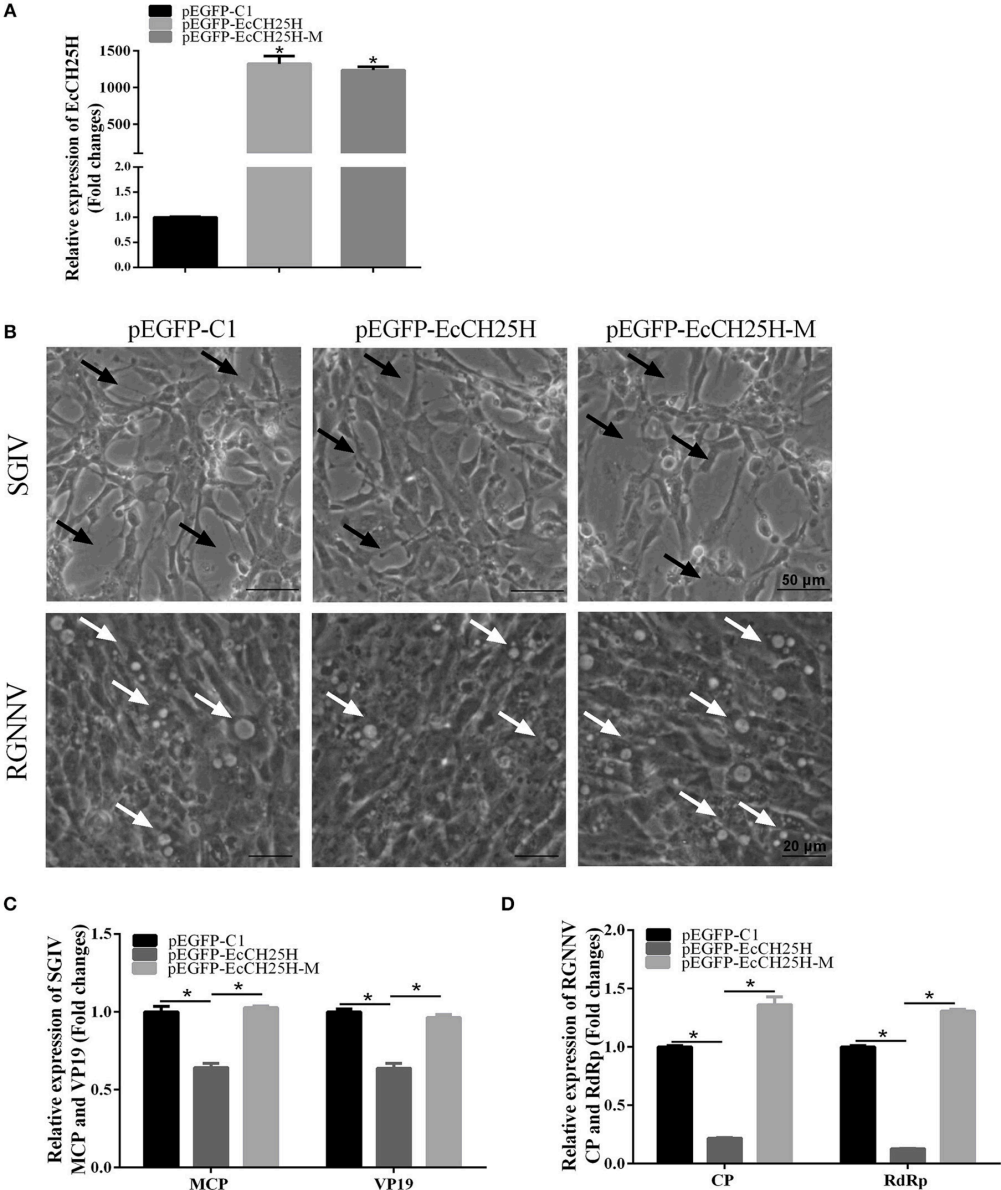
Overexpression of EcCH25H significantly inhibited SGIV and RGNNV replication. **(A)** Transcription of EcCH25H in EcCH25H- and EcCH25H-M-overexpressing cells. GS cells were transfected with pEGFP-EcCH25H or pEGFP-EcCH25H-M, respectively, and were collected to detect the transcription level of EcCH25H by qPCR at 48 h post-transfection. The primers EcCH25H-RT-F/R were designed to detect expression of EcCH25H and EcCH25H-M. **(B)** EcCH25H overexpression weakened the severity of CPE induced by RGNNV and SGIV in GS cells. GS cells were transfected with pEGFP-C1, pEGFP-EcCH25H, or pEGFP-EcCH25H-M for 24 h, and infected with SGIV or RGNNV, respectively. At 24 h.p.i., GS cells were observed for CPE using microscopy. In SGIV-infected cells, the black arrows indicated the severity of CPE induced by SGIV infection, which was characterized by cell rounding and aggregation of cells. The white arrows showed that vacuoles were induced by RGNNV infection. **(C,D)** Viral gene transcription of SGIV or RGNNV in EcCH25H- or EcCH25H-M-overexpressing cells. EcCH25H- and EcCH25H-M-overexpressing cells were infected with RGNNV or SGIV, and collected at 24 h.p.i. to determine by qPCR expression of MCP and VP19 of SGIV and CP and RdRp of RGNNV (*n* = 3, means ± SD). ^*^P < 0.05.

To explore the effects of EcCH25H overexpression on fish virus replication and whether it depends on the enzymatic activity of CH25H, GS cells were transfected with pEGFP-C1, pEGFP-EcCH25H, or pEGFP-EcCH25H-M for 24 h, and then infected with SGIV or RGNNV for a further 24 h. The transcription level of EcCH25H was examined by qPCR to confirm the successful ectopic expression of EcCH25H and EcCH25H-M in transfected cells. mRNA expression of EcCH25H and EcCH25H-M was significantly increased up to 1,322.2- and 1,235.7-fold compared with that of the control vector cells (Figure 4A). Compared to the control vector cells, the cytopathic effect (CPE) induced by SGIV and RGNNV was weakened in EcCH25H-overexpressing cells. In contrast, CPE severity was significantly increased in EcCH25H-M-overexpressing cells compared with EcCH25H-overexpressing cells (Figure 4B). Consistently, the transcription levels of the SGIV major capsid protein (MCP) and VP19 genes, as well as RGNNV CP and RdRp genes, were significantly inhibited when EcCH25H was overexpressed. However, the mutant significantly decreased the inhibitory effects of EcCH25H on viral gene transcriptions (Figures 4C,D). Thus, the results demonstrated that EcCH25H overexpression inhibited replication of RGNNV and SGIV, and that the antiviral effects were dependent on its enzymatic activity.

**Figure 5:**
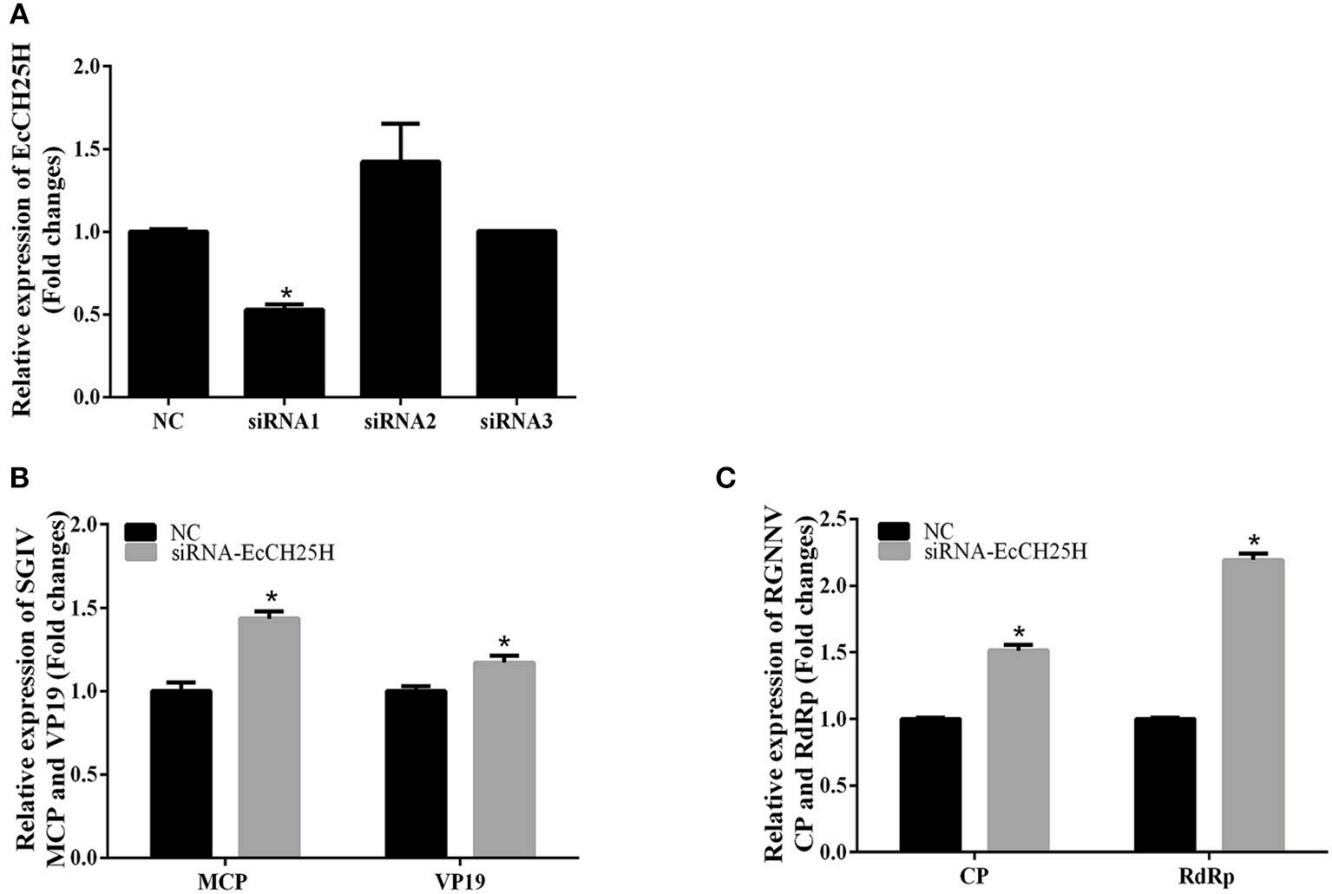
Knockdown of EcCH25H by siRNA promoted SGIV and RGNNV replication *in vitro*. **(A)** GS cells were transfected with siRNAs targeting EcCH25H or negative control siRNA (NC). At 48 h post-transfection, the CH25H mRNA levels were determined by qPCR. **(B,C)** Viral gene transcription of SGIV or RGNNV in siRNA-EcCH25H- and negative control siRNA (NC)-overexpressing cells. CH25H knockdown and control cells were infected with SGIV **(B)** or RGNNV **(C)** at 24 h, and were collected at 24 h.p.i. to determine expression of MCP and VP19 of SGIV and CP and RdRp of RGNNV (*n* = 3, means ± SD). ^*^P < 0.05.

To further investigate whether knockdown of EcCH25H promoted SGIV and RGNNV replication, we designed three siRNAs targeting EcCH25H, and examined interference efficiency in GS cells using qPCR. Compared with the negative control siRNA, siRNA1 decreased expression of EcCH25H, with 47% knockdown efficiency (Figure 5A). After transfection with siRNA-EcCH25H for 24 h, GS cells were infected with SGIV and RGNNV for a further 24 h, and collected to examine the transcription of viral genes by qPCR. Knockdown of EcCH25H by siRNA promoted SGIV and RGNNV replication compared with the cells transfected with the negative control siRNA (Figures 5B,C). Thus, it was proposed that EcCH25H exerted the antiviral effects on SGIV and RGNNV infection.

**Figure 6:**
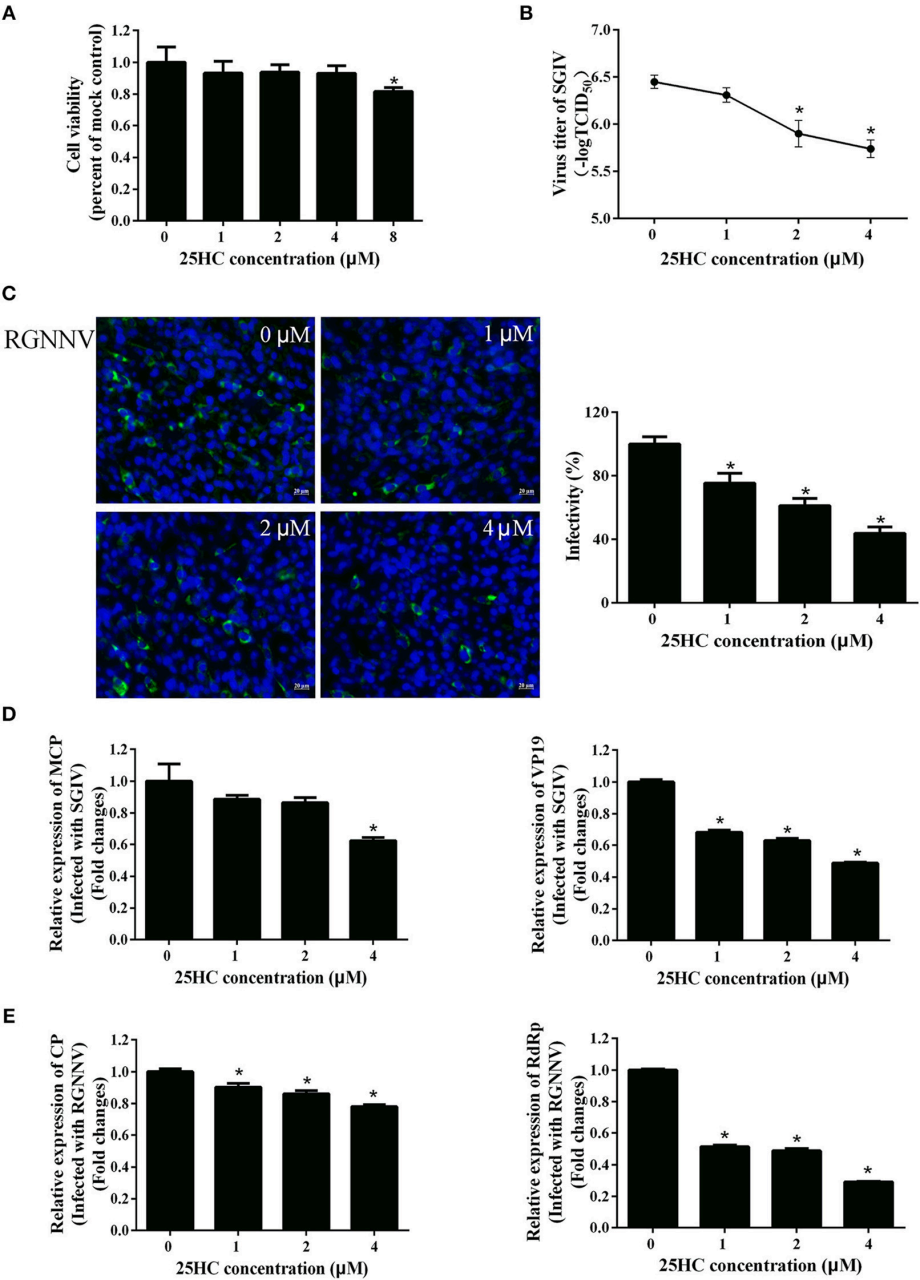
25HC inhibited SGIV and RGNNV replication *in vitro*. **(A)** Cell viability assay. GS cells were pretreated with chloroform (control) or 25HC at 1, 2, 4, or 8 μM. At 72 h post-treatment, cell viability was determined using an MTT assay. **(B–E)** 25HC inhibited SGIV and RGNNV infection. GS cells were pretreated with various concentrations of 25HC or chloroform for 12 h prior to SGIV or RGNNV infection, then the infected cells were analyzed at 24 h p.i. by immunofluorescence assay (IFA) using anti-CP antibody (green). The infectivity was quantified as the percentage of treated cells with incorporated viruses relative to that for control cells. The viral infectivity of control cells was set as 100% **(C)**. Meanwhile, GS cells were harvested for TCID_50_ assay of SGIV **(B)** and qPCR for expression of SGIV **(D)** or RGNNV **(E)** genes at 24 h.p.i. (*n* = 3, means ± SD). ^*^P < 0.05.

It has been reported that CH25H exerts antiviral activities by producing 25HC (18, 19, 41). To explore whether 25HC plays a role in SGIV or RGNNV infection, GS cells were pretreated with different concentrations of 25HC for 12 h, then infected with SGIV or RGNNV for 24 h. Cell viability was not decreased when cells were exposed to 4 μM 25HC for 72 h (Figure 6A). Virus titer assay indicated that 25HC significantly inhibited SGIV replication (Figure 6B). 25HC significantly inhibited expression of the CP protein of RGNNV in a dose-dependent manner (Figure 6C). 25HC significantly decreased the transcriptional level of MCP and VP19 of SGIV and CP and RdRp of RGNNV compared with chloroform (Figures 6D,E). Taken together, these results suggested that EcCH25H exhibited antiviral activity by producing 25HC, with the virus inhibition being dependent on the EcCH25H enzymatic activity.

### EcCH25H Overexpression Positively Regulates the IFN Immune and Inflammatory Response

**Figure 7:**
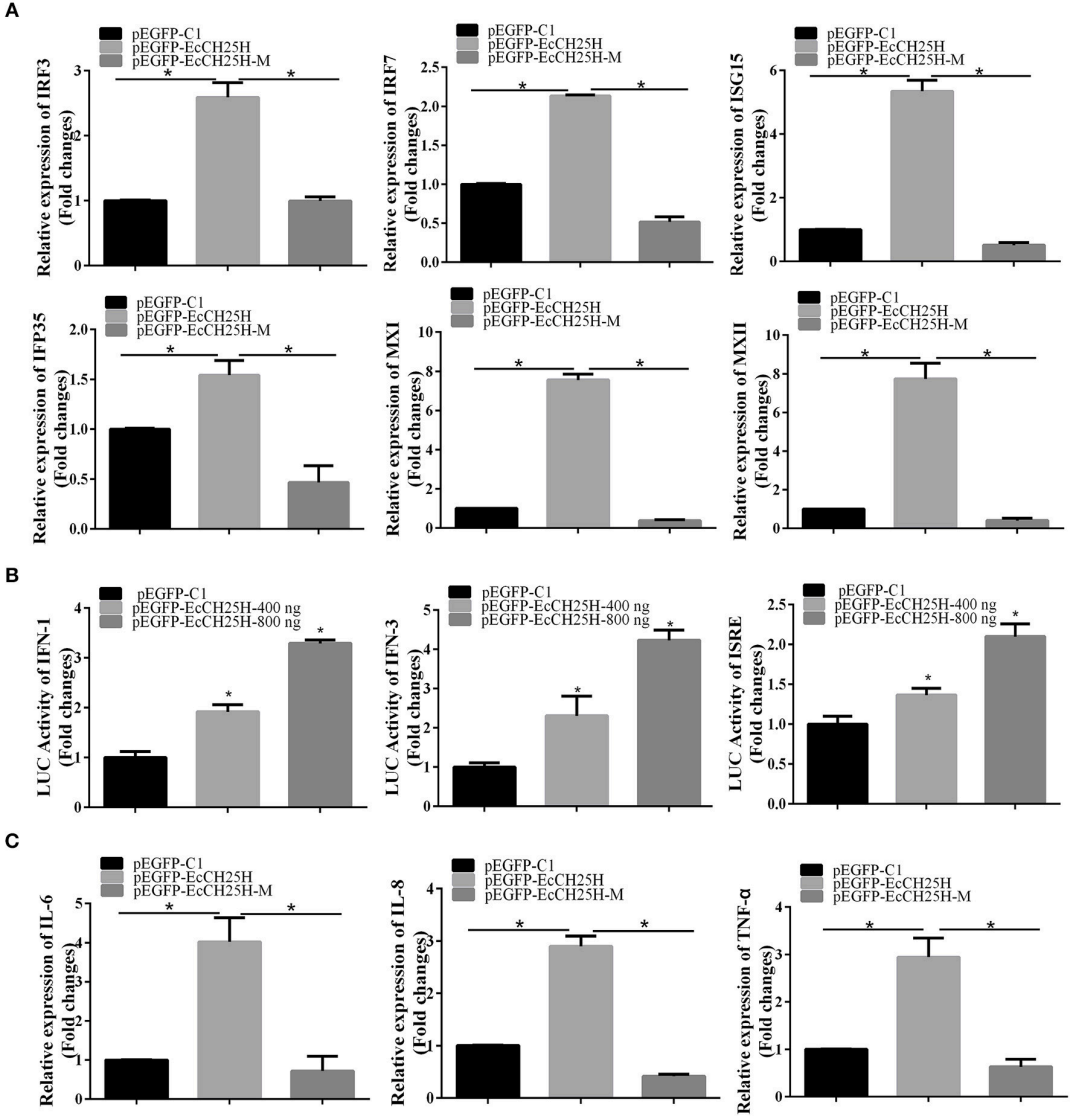
Overexpression of EcCH25H positively regulated IFN and inflammatory response. GS cells were transfected with pEGFP-EcCH25H or pEGFP-EcCH25H-M, and were collected at 48 h to detect the expression of IFN related signaling molecules **(A)** including IRF3, IRF7, ISG15, IFP35, MXI, MXII, and proinflammatory factors **(C)** including IL-6, IL-8, and TNF-α in pEGFP-EcCH25H-, pEGFP-EcCH25H-M- or pEGFP-C1-overexpressing cells by qPCR. Besides, GS cells were co-transfected with ISRE-Luc, IFN1-Luc, IFN3-Luc, and pEGFP-EcCH25H for 48 h, respectively, and cells were harvested to measure the luciferase activities of IFN-1, IFN-3, and ISRE promoter induced by EcCH25H using reporter gene assay **(B)** (*n* = 3, means ± SD). ^*^P < 0.05.

To clarify the effects of EcCH25H on host IFN immune and inflammatory response, we examined expression of IFN signaling molecules, and proinflammatory cytokines by qPCR and the promoter activities of IFN-1, IFN-3 and ISRE in EcCH25H- and EcCH25H-M-overexpressing cells. Compared with the controls, overexpression of EcCH25H significantly increased expression of several IFN-related genes, including IRF3, IRF7, ISG15, IFN-induced 35-kDa protein (IFP35), myxovirus resistance gene (MX)I and MXII. In EcCH25H-M-overexpressing cells, the concentration of mRNA transcripts was significantly lower than that in EcCH25H overexpressing cells (Figure 7A). Reporter gene analysis showed that EcCH25H overexpression significantly increased the luciferase activity of ISRE, IFN1, and IFN3 promoters in a dose-dependent manner (Figure 7B). We propose that EcCH25H positively regulated IFN immune response.

The effects of EcCH25H overexpression on expression of proinflammatory cytokines were also examined. mRNA production by tumor necrosis factor (TNF)-α, interleukin (IL)-6 and IL-8 genes was significantly increased in EcCH25H-overexpressing cells compared with control cells, with the stimulatory effects of EcCH25H being dependent on enzymatic activity (Figure 7C). Ectopic expression of EcCH25H *in vitro* up-regulated the expression of IFN and the inflammatory response, and was related to enzyme activity.

### EcCH25H Inhibits SGIV and RGNNV Entry

**Figure 8:**
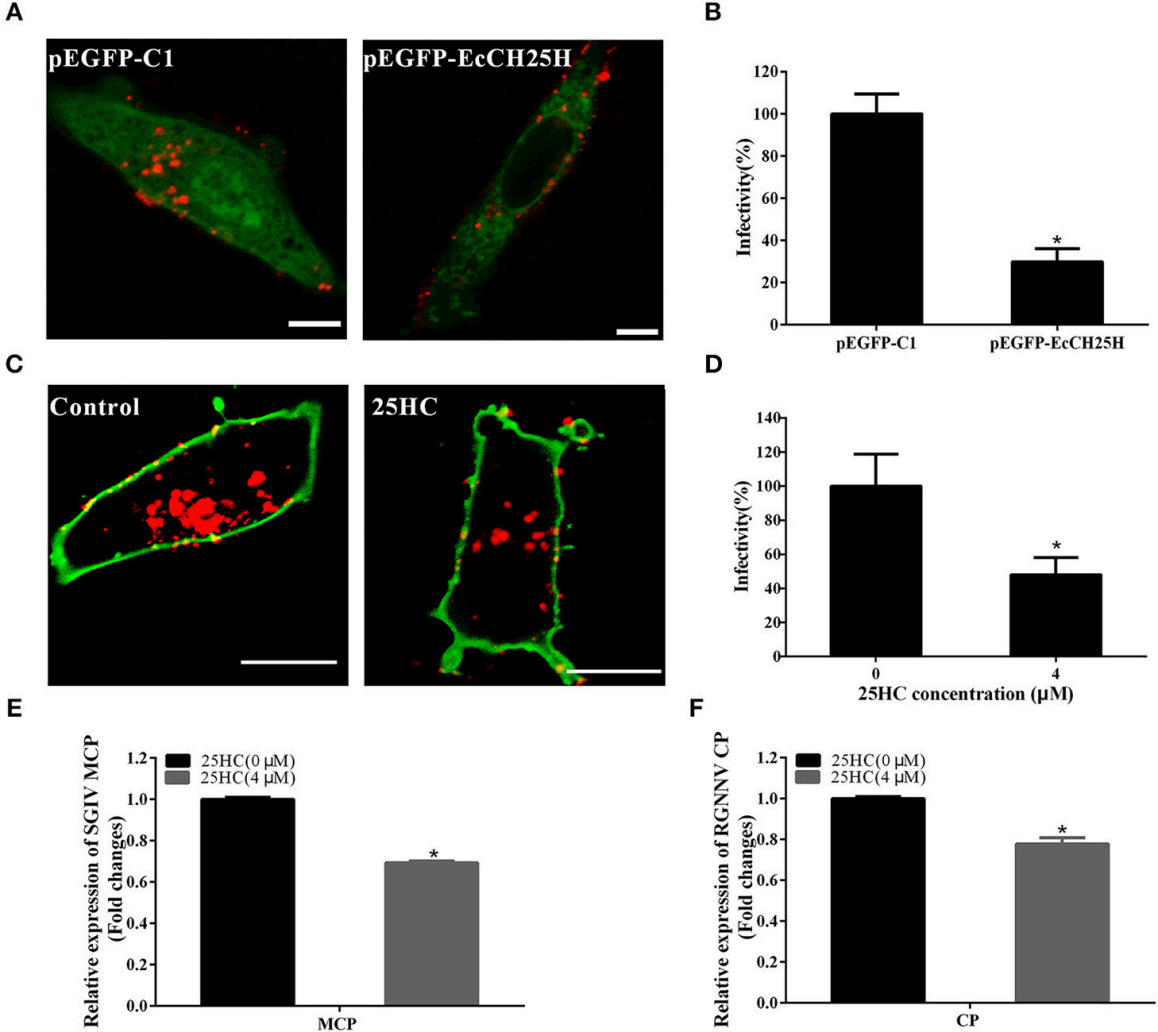
EcCH25H inhibited SGIV and RGNNV entry. **(A)** EcCH25H-overexpression inhibited SGIV uptake. GS cells were transfected with pEGFP-EcCH25H or pEGFP-C1 for 24 h, followed by infection with Cy5-labeled SGIV (red) for 1 h, and the cells were examined by CLSM. **(B)** EcCH25H overexpression decreased SGIV infectivity. **(C)** 25HC suppressed SGIV uptake. GS cells were pretreated with 25HC at 4 μM or chloroform (control) for 12 h, then infected with SGIV for 1 h and labeled with DiO (green). **(D)** 25HC decreased SGIV infectivity. The level of infection of the treated cells was normalized to that of the untreated cells. Scale bars were 20 μm. The data were represented as the means ± SEM from three independent experiments (*n* = 30). **(E,F)** GS cells were pretreated with chloroform or 25HC at 4 μM for 12 h, followed by SGIV (MOI = 8) or RGNNV (MOI = 8) infection. The relative expression of SGIV **(E)** or RGNNV **(F)** genes were quantified by qPCR (*n* = 3, means ± SD). ^*^P < 0.05.

To further investigate whether EcCH25H suppressed SGIV or RGNNV entry, GS cells were transfected with pEGFP-EcCH25H or pretreated with 25HC at 4 μM for 12 h, then infected with RGNNV or Cy5-labeled SGIV. EcCH25H overexpression reduced the number of red-fluorescence-labeled SGIV particles in the cytoplasm to 29.7% of that in empty-vector-transfected cells (Figures 8A,B). Furthermore, SGIV entry into GS cells pretreated with 25HC was significantly reduced to 48% of that in cells treated with chloroform (Figures 8C,D). 25HC decreased the transcriptional level of MCP of SGIV and CP of RGNNV compared with that achieved in cells exposed to chloroform (Figures 8E,F). These results suggested that EcCH25H and 25HC might influence the entry of SGIV or RGNNV, by reducing the infectivity of virus particles in the cytoplasm.

## Discussion

An increasing number of studies have shown that CH25H can inhibit various enveloped and non-enveloped viruses by production of 25HC (18, 23, 39). However, few have focused on the function of CH25H in lower vertebrates. Here, we cloned and characterized EcCH25H and analyzed its enzymatic product 25HC as antiviral restriction factors against iridovirus and nodavirus.

Based on bioinformatics analysis, we found that EcCH25H from *E. coioides* shared 86% identity to that from *L. crocea*. Amino acid alignment analysis has shown that EcCH25H contains the characteristic FA hydroxylase domain, which is similar to that of the zebrafish CH25H (42), suggesting that CH25H is conserved from lower vertebrates to mammals. Using qPCR analysis, our data showed that the concentration of EcCH25H increased significantly during SGIV and RGNNV infection, a finding which was consistent with the previous studies in which CH25H was induced in response to VSV (19), Zika virus (39), HCV (17), and MCMV (43). In addition, studies had demonstrated that CH25H could be induced by poly (I:C) (17) and LPS (44, 45), which was similar to the results of the present study, suggesting that CH25H is induced by PAMPs and plays a crucial role in the innate immune response against virus infection. In addition, subcellular localization analysis has shown that EcCH25H and the catalytically inactive mutant EcCH25H-M are both localized in the cytoplasm and have diffuse intracellular expression patterns, which are the same as the localization pattern of pig CH25H *in vitro* (21).

As mentioned above, the broad antiviral effects of CH25H have been demonstrated in mammalian cells for some viruses, and these studies have indicated that CH25H exerts an antiviral function by production of 25HC (39, 46). It is suggested that 25HC directly inhibits virus replication by suppressing viral gene expression (19, 43). In our study, ectopic expression of EcCH25H significantly weakened the severity of CPE induced by SGIV and RGNNV infection. Consistently, transcription of viral genes was inhibited by EcCH25H overexpression and by treatment with 25HC, but was enhanced by silencing EcCH25H. In other words, EcCH25H and 25HC suppressed viral gene expression to inhibit infection of SGIV and RGNNV.

In addition, recent studies have shown that CH25H-M lacking enzymatic activity to produce 25HC still had antiviral activity against HCV (19) and PRRSV (18), but not against other viruses, such as mouse hepatitis virus 68 and PRV (21), suggesting that CH25H can inhibit viral infection through either enzyme-activity-dependent or -independent pathways. In this study, we found that EcCH25H-M was unable to inhibit replication of SGIV and RGNNV, confirming that the antiviral mechanism of EcCH25H against SGIV and RGNNV is dependent on its enzyme activity and similar to that against other viruses, such as HCV (19) and PRRSV (18). Unexpectedly, the EcCH25H-M showed no effect on SGIV, but enhanced RGNNV replication moderately, indicating that the enhancement of virus replication by EcCH25H-M might be dependent on virus type.

Moreover, as a naturally occurring secreted oxysterol, 25HC can block the fusion of the enveloped viruses with cell membranes to inhibit viral entry (47). For example, 25HC can modify cell membranes to inhibit efficient fusion between HIV and cell membranes, without affecting HIV transcription, translation and budding (19). 25HC inhibits VSV entry, by a route which is independent of regulation of SREBP2, mevalonate production, or protein prenylation (19). In addition, 25HC inhibits HCV infection at a post-entry stage by suppressing activation of SREBP2 (17). Our results show that EcCH25H overexpression and 25HC treatment inhibit SGIV entry, in agreement with previous observation for VSV (19). As an enveloped virus, SGIV entered host cells by fusion with the plasma membrane, and EcCH25H might inhibit SGIV entry by blocking the fusion of viral envelope with cell membranes. Differently, as a non-enveloped virus, RGNNV entry might be affected by the action of EcCH25H on interferon immune response. The detailed mechanism by which EcCH25H suppresses SGIV or RGNNV entry needed further investigation.

A growing number of studies have revealed that CH25H is involved in IFN immune response in few mammals (17, 21, 24). 25HC promotes the secretion of some inflammatory cytokines, such as IL-6, IL-8, and macrophage colony-stimulating factor in macrophages and epithelial cells (48, 49). To clarify the effects of EcCH25H on host IFN and inflammatory response, we examined the expression of IFN signaling molecules and proinflammatory cytokines in EcCH25H- and EcCH25H-M-overexpressing cells. Expression levels of several IFN-related cytokines, including IRF3, IRF7, ISG15, IFP35, MXI, and MXII, as well as that of inflammatory cytokines, such as TNF-α, IL-6, and IL-8, were all significantly increased. Further analysis showed that EcCH25H-overexpression enhanced IFN and ISRE promoter activities. Thus, we speculate that EcCH25H positively regulates IFN immune and proinflammatory response to inhibit SGIV and RGNNV infection.

In summary, we demonstrated the roles of EcCH25H in RGNNV and SGIV replication and entry. A fish CH25H homolog from orange-spotted grouper (EcCH25H) was cloned and characterized. EcCH25H encoded a cytoplasmic protein and was induced by RGNNV or SGIV infection, or by treatment with either LPS or poly (I:C). Overexpression of EcCH25H *in vitro* significantly suppressed replication of SGIV and RGNNV due to its positive effect on the host IFN immune response, which is contrary to the results of RNAi. Furthermore, our results indicate that EcCH25H inhibits SGIV and RGNNV entry, and shed more light on the mode-of-action of CH25H against fish virus infection.

## Author Contributions

YZ performed the experiments, analyzed the data, and wrote the manuscript. LW and XH prepared virus stocks including SGIV, RGNNV and the purified SGIV. SW participated in confocal microscopy analysis. YH and QQ participated in the design of the study, data analysis and revised the manuscript.

### Conflict of Interest Statement

The authors declare that the research was conducted in the absence of any commercial or financial relationships that could be construed as a potential conflict of interest.
